# Frequent users of emergency departments and patient flow in Alberta and Ontario, Canada: an administrative data study

**DOI:** 10.1186/s12913-020-05774-6

**Published:** 2020-10-12

**Authors:** Anqi Chen, Scott Fielding, X. Joan Hu, Patrick McLane, Andrew McRae, Maria Ospina, Rhonda J. Rosychuk

**Affiliations:** 1 Department of Pediatrics, Faculty of Medicine & Dentistry, University of Alberta, 3-524 Edmonton Clinic Health Academy, Edmonton, Alberta T6G 1C9 Canada; 2 Emergency Strategic Clinical Network, Alberta Health Services, Edmonton, Alberta T5J 3E4 Canada; 3 Department of Statistics and Actuarial Science, Simon Fraser University, Burnaby, British Columbia V5A 1S6 Canada; 4 Department of Emergency Medicine, University of Alberta, Edmonton, Alberta T6G 2R7 Canada; 5 Department of Emergency Medicine, University of Calgary, Calgary, Alberta T2N 2T9 Canada; 6 Department of Community Health Sciences, University of Calgary, Calgary, Alberta T2N 4Z6 Canada; 7 Department of Obstetrics & Gynecology, University of Alberta, Edmonton, Alberta T6G 2S2 Canada; 8 School of Public Health, University of Alberta, Edmonton, Alberta T6G 1C9 Canada; 9 Department of Mathematical and Statistical Sciences, University of Alberta, Edmonton, Alberta T6G 2G1 Canada

**Keywords:** Emergency department, Frequent health service users, Patient flow

## Abstract

**Background:**

This paper describes and compares patient flow characteristics of adult high system users (HSUs) and control groups in Alberta and Ontario emergency departments (EDs), Canada.

**Methods:**

Annual cohorts of HSUs were created by identifying patients who made up the top 10% of ED users (by count of ED presentations) in the National Ambulatory Care Reporting System during 2011–2016. Random samples of patients not in the HSU groups were selected as controls. Presentation (e.g., acuity) and ED times (e.g., time to physician initial assessment [PIA], length of stay) data were extracted and described. The length of stay for 2015/2016 data was decomposed into stages and Cox models compared time between stages.

**Results:**

There were 20,343,230 and 18,222,969 ED presentations made by 7,032,655 and 1,923,462 individuals in the control and HSU groups, respectively. The Ontario groups had higher acuity than the Alberta groups: about 20% in the Ontario groups were from the emergent level whereas Alberta had 11–15%. Time to PIA was similar across provinces and groups (medians of 60 min to 67 min). Lengths of stay were longest for Ontario HSUs (median = 3 h) and shortest for Alberta HSUs (media*n* = 2.2 h). HSUs had shorter times to PIA (hazard ratio [HR] = 1.03; 95% confidence interval [CI] 1.02,1.03), longer times from PIA to decision (HR = 0.84; 95%CI 0.84,0.84), and longer times from decision to leaving the ED (HR = 0.91; 95%CI 0.91,0.91).

**Conclusions:**

Ontario HSUs had higher acuity and longer ED lengths of stay than the other groups. In both provinces, HSU had shorter times to PIA and longer times after assessment.

## Background

Frequent users of emergency departments (EDs) are a small number of patients who are responsible for a large proportion of ED presentations [1]. No uniformly accepted definition of a frequent ED user has been identified. Some authors have used definitions such as ≥4 [2], ≥5 [3], and ≥ 7 [4] within a 12 month period. Whatever the definition, frequent users are an important group to of patients because some frequent users may be able to receive more appropriate care in other health settings [5] (e.g. settings more equipped for the management of chronic conditions) and some believe these patients contribute to ED crowding [6].

A systematic review of adult frequent ED users in the United States (US) indicates that frequent ED users are disproportionately sicker and were more likely to be from younger age groups, have public insurance, and be female [5, 7]. For EDs outside the US, frequent ED users were more likely to be younger and have chronic illnesses compared to non-frequent ED users [8]. Significant associations have also been reported between frequent ED users and asthma [9], psychological distress [10, 11], and substance use [4, 12]. Other studies have suggested that a misunderstanding of medical necessity and access issues (e.g., ease, timing, geography) are reasons that frequent users present to EDs [13]. In terms of outcomes, higher mortality, hospital admissions, and outpatient visits have been reported for frequent ED users compared to non-frequent users [14]. Frequent ED users seem to be high users of health care generally [1, 7].

Previous studies generally focus on one ED or EDs within one administrative jurisdiction and often target a specific diagnosis. We focus on cohorts created from two provinces in Canada, Alberta and Ontario, and presentations for any health condition. Despite the key role of the ED in health care delivery, research and surveillance opportunities in this setting, while occasionally employed, are clearly underutilized, especially in Canada. There is a paucity of research on ED presentations in Canada, especially for ambulatory care sensitive conditions, and on frequent ED users. A better understanding of frequent ED users and their impact on EDs and the health care system can lead to targeted approaches that help reduce frequent ED use without compromising care.

We examine key duration outcomes recommended for EDs [15]: time to physician initial assessment (PIA) and length of stay ED (LOS). The primary objectives of this study were to describe and compare outcomes between high system users (HSUs) of EDs and control groups in Alberta and Ontario as well as to compare HSUs between provinces. These comparisons are also made by acuity level as acuity level is an important determinant of patient flow through the ED. A secondary objective was to deconstruct the flow through the ED and determine factors affecting the time in each stage of a presentation.

## Methods

### Study design

This retrospective cohort study used population-based health administrative databases from the provinces of Alberta and Ontario, Canada during April 1, 2011, to March 31, 2016. This study was approved by the University of Alberta Health Research Ethics Board. The funding organization did not have input in the conduct and reporting of the study.

### Study setting and population

Provinces in Canada have uniform single-payer health systems that are administrated by individual provinces to provide medically necessary health care. The western Canadian province of Alberta has > 4 million residents and the central Canadian province of Ontario has > 14 million residents [16].

The Dynamic Cohort of Complex, High System Users [16] – based on acute care cost, highest length of stay, most frequent hospitalizations, and most frequent ED presentations – has been created as a collaboration between the Canadian Institutes of Health Research and the Canadian Institute for Health Information (CIHI). Alberta and Ontario are the only two provinces in Canada that report on all ED presentations to the National Ambulatory Care Reporting System (NACRS) [17] and CIHI used this database to extract and link the data needed for our study.

The study population consists of individuals aged ≥18 years at the end of the fiscal year in the HSU or control groups who presented to EDs during the study period. A dynamic cohort of the most frequent users was created for each province and fiscal year by identifying the top 10% of patients with respect to the number of ED presentations [16]. These patients form the HSU group. Control groups were also created for each province and fiscal year by selecting a random sample of patients not in the HSU group using a sampling ratio of 4:1 [16]. These design choices were made by CIHI. The number of presentations was based on unscheduled presentations; however, our extract has included both scheduled and unscheduled presentations as there may be variability in coding and relatively few (< 1%) ED presentations were classified as scheduled.

### Study protocol

The NACRS database provides data on characteristics of ED presentations including dates and times, triage level, and diagnoses. The demographic data include age in years at the date of ED presentation, sex, and the forward sortation area (first three characters of the postal code) of residence to provide a proxy for urban and rural location of residence. The full postal code was used by CIHI to obtain “as the crow flies” distances from patients’ homes to the hospital they visited (kilometres [km]). For Ontario, the demographic data include access to primary health care (e.g., family physician, other, none). The date/time variables included the date/time of registration, triage, physician initial assessment, disposition decision, and patient leaving the ED. The start of the presentation (registration) defined the fiscal year, month of year, weekday/weekend, and time of shift (daytime 08:00–15:59, evening 16:00–23:59, night 00:00–07:59). Mode of arrival included type of ambulances and no ambulance arrival. Triage level represents the urgency of ED care required by the individual and is based on the Canadian Emergency Department Triage and Acuity Scale (CTAS) [18]. The triage codes are resuscitation (1), emergency (2), urgent (3), semi-urgent (4), and non-urgent (5).

Diagnoses are provided as International Classification of Diseases (ICD-10-CA) [19] codes with up to 10 codes recorded. The main diagnosis code was classified into diagnosis categories using a combination of the Quan et al. adaptation [20] to the Deyo/Charlson comorbidity coding scheme (R package icd by Wasey, J O and R Core Team) and the classification of diagnoses from Guttmann et al. [21]. Any overlapping ICD codes were kept only in the Quan et al. coding scheme to ensure mutually exclusive diagnosis categories.

Patients are given one of 15 disposition codes according to how they are released from ED and we have grouped these as discharges (i.e., discharged home without support services, discharged to place of residence with support services), admissions (admitted into reporting facility as an inpatient to critical care unit or operating room, admitted into reporting facility as inpatient [to another unit]), transfers (transferred to another acute care facility, transferred to another non-acute care facility, intra-facility transfer to day surgery, intra-facility transfer to the emergency department, intra-facility transfer to clinic), deaths (death after arrival; death on arrival), left without being seen (LWBS; left without being seen [not triaged], left without being seen [triaged]), and left against medical advice (LAMA; left without treatment [triaged], left after triage and initiation of treatment).

### Key outcome measures

The main study outcomes were time to PIA and LOS calculated for HSU and control groups in each province. These outcomes are recommended as national benchmarks for ED for ED performance [15]. Time to PIA was calculated as the difference between physician initial assessment and the start of the ED presentation. All patients would be seen by a physician unless the patient left without being seen. If a patient did see a physician and the time to PIA was missing, the time was interval-censored between the start and disposition decision times. The ED LOS is also provided and depends on disposition: it is calculated as the time from the start of ED presentation until the time of the disposition decision for discharged patients or time the patient left the ED for admitted patients [22]. For discharged patients, the time of disposition and the time of leaving the ED are essentially the same.

### Data analysis

Data cleaning included combining overlapping ED presentations for a patient into a single presentation and considering durations > 12 h for time to physician initial assessment and > 7 days for time to disposition decision, time to patient leaving the ED, and ED LOS as missing (presumed inaccurate). Numerical summaries (i.e., means, medians, standard deviations [SDs], IQR represented as [25th percentile, 75th percentile]) and counts (percentages) describe patient demographics and ED presentation characteristics. When times were missing, Kaplan-Meier estimates of the median time and associated 95% confidence intervals (CIs) were also provided. Data are summarized by province and by HSU cohort status. To obtain 95% Is for key outcomes, the cluster bootstrap with 500 samples was used to adjust for the correlated data from the same individual. Further, Cox models with province, group, and CTAS level as predictors were obtained for times to transition along the key stages within the overall ED presentation for the 2015/2016 data (Supplementary Fig. 1, Additional File 1) similar to Liu et al. [23]. These models assume the ED presentations are independent for computational feasibility. Hazard ratios (HRs) and associated 95% confidence intervals (CIs) are provided. Diagnostic plots were used to check the proportional hazards and other assumptions: model assumptions were not violated. All analyses were conducted in R (Vienna, Austria; Version 3.5.1) [24].

## Results

### Characteristics of study subjects and ED presentations

During the entire study period, there were 20,343,230 and 18,222,969 ED presentations made by 7,032,655 and 1,923,462 individuals in the control and HSU groups, respectively. As the definition of HSU was based on the fiscal year, different patients may have comprised the HSU and control groups over a fiscal year. The proportion of adults that were HSUs in each of the five years was 1.1% in Alberta and 3.8% in Ontario. Table 1 provides the basic demographic summaries and the number of ED presentations by group and fiscal year.

**Table 1:** Basic demographics and ED presentations by province, group, and fiscal year

Fiscal Year	Characteristic	Alberta		Ontario	
		Control	HSU	Control	HSU
2011/2012	Number of patients	390,247	97,736	1,743,552	436,438
	Female (%)	294,575 (51.8)	368,229 (54.4)	1,167,536 (53.2)	1,108,572 (54.9)
	Male (%)	274,551 (48.2)	308,330 (45.6)	1,028,548 (46.8)	909,660 (45.1)
	Age at ED presentation				
	Mean (SD)	45.4 (19.3)	48.0 (20.2)	48.7 (19.9)	50.6 (21.0)
	Median (Q1, Q3)	43.0 (29.0, 58.0)	46.0 (30.0, 63.0)	48.0 (32.0, 63.0)	49.0 (32.0, 68.0)
	Number of ED presentations	569,126	676,559	2,196,106	2,018,238
	ED presentations per patient				
	Mean (SD)	1.5 (0.7)	6.9 (6.8)	1.3 (0.5)	4.6 (3.7)
	Median (Q1, Q3)	1.0 (1.0, 2.0)	5.0 (4.0, 7.0)	1.0 (1.0, 2.0)	4.0 (3.0, 5.0)
2012/2013	Number of patients	404,055	101,419	1,790,443	448,316
	Female (%)	299,402 (51.7)	379,561 (54.6)	1,182,717 (52.9)	1,138,327 (55.0)
	Male (%)	280,075 (48.3)	315,128 (45.4)	1,050,996 (47.1)	929,524 (45.0)
	Age at ED presentation				
	Mean (SD)	45.4 (19.1)	48.1 (20.2)	48.9 (19.8)	51.1 (21.2)
	Median (Q1, Q3)	43.0 (29.0, 58.0)	46.0 (30.0, 63.0)	48.0 (32.0, 63.0)	50.0 (32.0, 68.0)
	Number of ED presentations	579,477	694,689	2,233,736	2,067,866
	ED presentations per patient				
	Mean (SD)	1.4 (0.7)	6.8 (6.8)	1.2 (0.5)	4.6 (3.8)
	Median (Q1, Q3)	1.0 (1.0, 2.0)	5.0 (4.0, 7.0)	1.0 (1.0, 1.0)	4.0 (3.0, 5.0)
2013/2014	Number of patients	406,584	102,268	1,794,311	453,011
	Female (%)	295,315 (51.2)	377,763 (54.4)	1,175,974 (52.8)	1,154,708 (55.1)
	Male (%)	281,447 (48.8)	316,690 (45.6)	1,050,875 (47.2)	939,564 (44.9)
	Age at ED presentation				
	Mean (SD)	45.5 (18.9)	48.0 (20.2)	49.0 (19.7)	51.2 (21.2)
	Median (Q1, Q3)	43.0 (30.0, 58.0)	46.0 (30.0, 63.0)	48.0 (32.0, 63.0)	50.0 (32.0, 68.0)
	Number of ED presentations	576,765	694,457	2,226,878	2,094,329
	ED presentations per patient				
	Mean (SD)	1.4 (0.7)	6.8 (6.5)	1.2 (0.4)	4.6 (3.9)
	Median (Q1, Q3)	1.0 (1.0, 2.0)	5.0 (4.0, 7.0)	1.0 (1.0, 1.0)	4.0 (3.0, 5.0)
2014/2015	Number of patients	411,570	103,594	1,750,579	467,402
	Female (%)	296,255 (51.3)	376,531 (54.0)	1,139,718 (52.6)	1,193,239 (55.2)
	Male (%)	281,673 (48.7)	321,089 (46.0)	1,025,303 (47.4)	969,462 (44.8)
	Age at ED presentation				
	Mean (SD)	45.8 (19.0)	48.3 (20.3)	49.3 (19.7)	51.7 (21.4)
	Median (Q1, Q3)	43.0 (30.0, 59.0)	47.0 (31.0, 64.0)	49.0 (32.0, 64.0)	51.0 (33.0, 69.0)
	Number of ED presentations	577,930	697,620	2,165,039	2,162,728
	ED presentations per patient				
	Mean (SD)	1.4 (0.7)	6.7 (6.6)	1.2 (0.4)	4.6 (3.9)
	Median (Q1,Q3)	1.0 (1.0, 2.0)	5.0 (4.0, 7.0)	1.0 (1.0, 1.0)	4.0 (3.0, 5.0)
2015/2016	Number of patients	401,923	101,250	1,714,037	478,424
	Female (%)	287,525 (51.3)	371,621 (54.1)	1,110,576 (52.5)	1,218,732 (54.8)
	Male (%)	273,228 (48.7)	315,196 (45.9)	1,003,345 (47.5)	1,003,386 (45.2)
	Age at ED presentation				
	Mean (SD)	46.1 (18.9)	48.8 (20.3)	49.5 (19.6)	51.8 (21.3)
	Median (Q1, Q3)	43.0 (30.0, 59.0)	47.0 (31.0, 64.0)	49.0 (33.0, 64.0)	51.0 (33.0, 69.0)
	Number of ED presentations	560,755	686,817	2,113,961	2,222,173
	ED presentations per patient				
	Mean (SD)	1.4 (0.6)	6.8 (7.0)	1.2 (0.4)	4.6 (4.1)
	Median (Q1, Q3)	1.0 (1.0, 2.0)	5.0 (4.0, 7.0)	1.0 (1.0, 1.0)	4.0 (3.0, 5.0)

The vast majority of ED presentations in each group and province did not involve an ambulance in 2015/2016 (79.9 to 88.1%, Table 2). However, more presentations from HSUs were by ambulance than controls. The Ontario HSU group had the most use of ground ambulance (20.1%) and the Alberta control group had the lowest use (11.8%, Table 2). Across provinces and groups, similar proportions were seen for type of day (weekday/weekend) and shift. The Ontario groups had higher acuity than the Alberta groups: 20.6 and 19.8% in the Ontario HSU and control groups, respectively, were from emergent level (CTAS 2) while 11.3 and 14.5% in the Alberta HSU and control groups, respectively. Triage levels over time (Supplementary Fig. 2, Additional File 1) show that the Ontario groups had similar acuity and the Alberta groups have more variability over time. The analogous Table 2 results for the other fiscal years were similar and are not shown.

**Table 2:** Emergency department presentation characteristics by province and group for 2015/2016

Characteristic	Alberta		Ontario	
	Control (*n* = 560,755)	HSU (*n* = 686,817)	Control (*n* = 2,113,961)	HSU (*n* = 2,222,173)
Admit via ambulance				
No ambulance arrival (e.g., walk in) (%)	494,292 (88.1)	588,648 (85.7)	1,803,287 (85.3)	1,774,770 (79.9)
Ground ambulance (%)	65,952 (11.8)	97,268 (14.2)	309,852 (14.7)	445,702 (20.1)
Air ambulance (%)	346 (0.1)	437 (0.1)	235 (0.0)	404 (0.0)
Any combination of ground, air, or water ambulance (%)	165 (0.0)	464 (0.1)	587 (0.0)	1297 (0.1)
Day				
Weekday (%)	401,925 (71.7)	505,314 (73.6)	1,523,089 (72.0)	1,628,069 (73.3)
Weekend (%)	158,830 (28.3)	181,503 (26.4)	590,872 (28.0)	594,104 (26.7)
Shift				
00:00–07:59 (%)	69,287 (12.4)	88,230 (12.8)	274,707 (13.0)	307,453 (13.8)
08:00–15:59 (%)	285,340 (50.9)	351,680 (51.2)	1,074,124 (50.8)	1,117,009 (50.3)
16:00–23:59 (%)	206,128 (36.8)	246,907 (35.9)	765,130 (36.2)	797,711 (35.9)
Triage level				
1 - Resuscitation (%)	3184 (0.6)	3093 (0.5)	19,235 (0.9)	20,660 (0.9)
2 - Emergent (%)	81,079 (14.5)	77,517 (11.3)	417,725 (19.8)	457,096 (20.6)
3 - Urgent (%)	214,526 (38.3)	220,567 (32.1)	967,426 (45.8)	1,011,687 (45.5)
4 - Less-urgent (Semi-urgent) (%)	205,098 (36.6)	238,190 (34.7)	639,120 (30.2)	593,952 (26.7)
5 - Non-urgent (%)	45,912 (8.2)	116,028 (16.9)	60,550 (2.9)	113,907 (5.1)
Missing / Unavailable (%)	10,956 (2.0)	31,422 (4.6)	9905 (0.5)	24,871 (1.1)
Time to physician initial assessment (PIA) (minutes)				
Median (Q1, Q3)*	67.0 (35.0, 121.0)	60.0 (30.0, 112.0)	67.0 (35.0, 119.0)	63.0 (32.0, 115.0)
Missing / N.A. (%)	113,512 (20.2)	264,046 (38.4)	156,479 (7.4)	243,843 (11.0)
Estimated median time (95% confidence interval) †	70 (70, 70)	63 (63, 63)	69 (69, 69)	65 (65, 65)
Time to disposition decision (hours)				
Median (Q1, Q3)	2.6 (1.4, 4.6)	2.2 (1.1, 4.4)	2.8 (1.6, 4.5)	2.9 (1.6, 5.0)
Missing / N.A. (%)	11,098 (2.1)	27,569 (4.4)	10,785 (0.5)	25,475 (1.2)
Estimated median time (95% confidence interval) †	2.6 (2.6, 2.7)	2.2 (2.2, 2.3)	2.8 (2.8, 2.8)	3.0 (3.0, 3.0)
Disposition				
Discharged (%)	478,332 (85.3)	575,360 (83.8)	1,826,261 (86.4)	1,789,388 (80.5)
Admitted (%)	51,136 (9.1)	65,087 (9.5)	197,128 (9.3)	291,669 (13.1)
Transferred (%)	10,445 (1.9)	16,438 (2.4)	24,262 (1.1)	44,444 (2.0)
Left without being seen (LWBS) (%)	14,604 (2.6)	19,542 (2.8)	49,749 (2.4)	71,189 (3.2)
Left against medical advice (LAMA) (%)	5685 (1.0)	10,179 (1.5)	13,955 (0.7)	24,364 (1.1)
Death (%)	553 (0.1)	211 (0.0)	2606 (0.1)	1119 (0.1)
Length of stay (hours)‡				
Median (Q1, Q3)	2.7 (1.4, 4.8)	2.3 (1.1, 4.7)	2.8 (1.6, 4.8)	3.1 (1.6, 5.5)
Missing (%)	10,983 (2.1)	27,406 (4.4)	3741 (0.2)	6829 (0.3)
Estimated median time (95% confidence interval) †	2.7 (2.7, 2.7)	2.3 (2.3, 2.3)	2.8 (2.8, 2.8)	3.1 (3.1, 3.1)

*n* number of emergency department presentations, *SD* standard deviation, *Q1* 25th percentile, *Q3* 75th percentile, *N.A.* not applicable, * missing times removed from calculation, *†* all data used and missing times interval censored, *‡* excluding left without being seen and left against medical advice

When the main diagnosis code was categorized (Supplementary Table 1, Additional File 1), injury and trauma had the highest proportion of ED presentations across all provinces and groups. Controls had more presentations for injury and trauma (around 35% in each province) than HSUs (19.1% in Alberta, 21.9% in Ontario). HSUs had about double the proportion of presentations for mental health reasons and diabetes than controls. Alberta HSUs had the highest proportion of diagnoses that were not categorized by either coding scheme (38.3% vs 21.5–27.7%). The rest of the diagnosis categories had similar proportions of ED presentations across provinces and groups.

The vast majority of presentations ended in discharge (Alberta: 85.3% control, 83.8% HSU; Ontario: 86.4% control, 80.5% HSU; Table 2). The Ontario HSU group had higher admissions (13.1%) than the control group and the Alberta groups. The Ontario HSU group also had the most LWBS (3.2%) whereas the Alberta HSU group had the most LAMA (1.5%) compared to the other groups. The HSU groups had higher LWBS and LAMA than their provincial control groups. There were relatively few deaths in any of the groups.

**Fig. 1:**
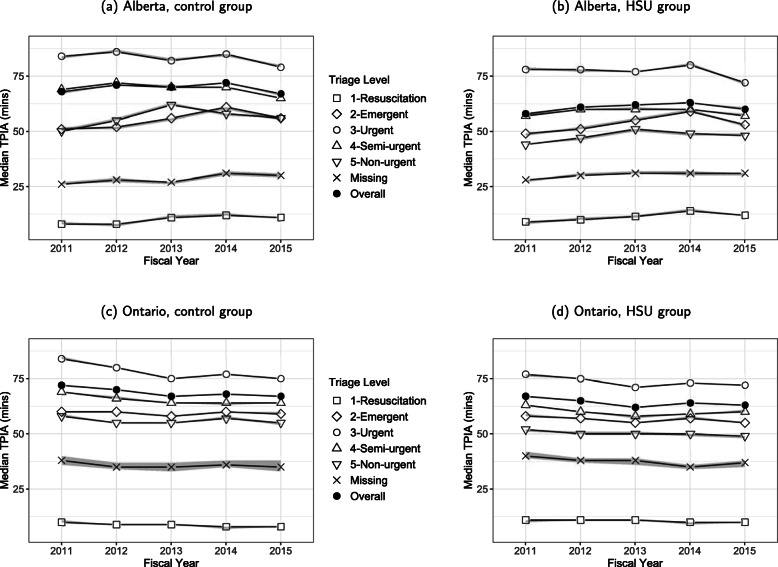
Median time to physician initial assessment by triage level, province, group, and fiscal year

Time to physician initial assessment was similar across provinces and groups (medians of 60 min to 67 min, Table 2). Notably, the Alberta HSUs had a large proportion of missing times to PIA (38.4%) followed by Alberta controls (20.2%), whereas the Ontario groups had missing at most 11%. The estimated median times that incorporated censoring for missing PIA provided almost identical medians as the sample medians of the non-missing data. When examined by time and triage level (Fig. 1), the median times were fairly stable over time and CTAS levels 2 to 5 had more similar median times in Ontario than compared to Alberta.

**Fig. 2:**
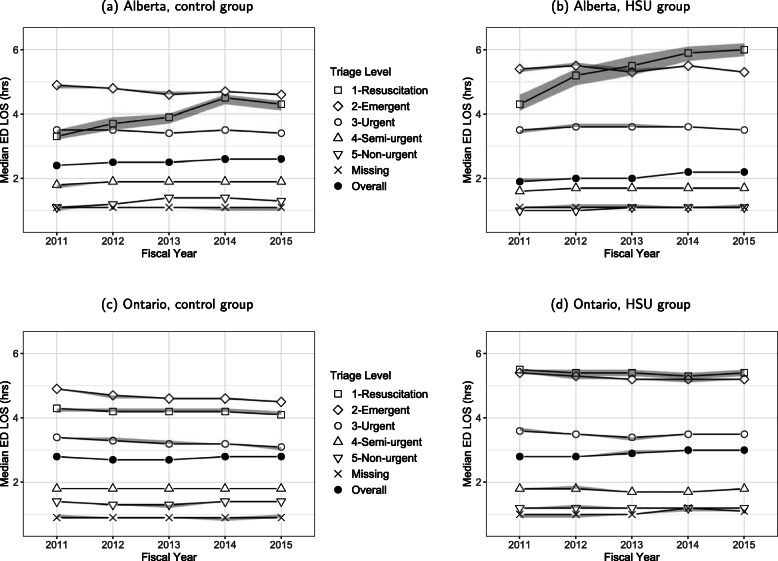
Median ED length of stay by triage level, province, group, and fiscal year

There were lower times to disposition decision and overall LOS in the Alberta groups compared to the Ontario groups in 2015/2016. The Ontario control group had median times to disposition decision and LOS about 10 min shorter than the HSU group. Conversely, the Alberta control group had median times to disposition decision about 25 min longer than the HSU group. In both provinces, the HSU groups had longer LOS compared with the control groups when the triage level was resuscitation or emergent (Fig. 2). The median LOS has remained stable over time in Ontario; however, in Alberta, the median LOS for presentations with resuscitations has increased over time in both the HSU and control groups.

When LOS was further examined by disposition (Supplementary Table 2, Additional File 1), the median LOS for presentations ending in discharge was 2hh30min for the Ontario control group, 2h36min for the Ontario HSU group, 2h24min for the Alberta control group, and 1h54min for the Alberta HSU group in 2015/2016. When triage level was considered in Supplementary Fig. 3, Additional File 1, the median LOS values for resuscitation and emergent presentations were longer compared with Ontario. Within Alberta, the HSU group had a longer median LOS value than the control group for those levels. For admissions/transfers in 2015/2016, the median LOS was 9h18min for the Ontario control and HSU groups, 8h18min for the Alberta control group, and 7h18min for the Alberta HSU group. Overall, Alberta times were shorter than Ontario times. The HSU group had lower times than the control for Alberta but not Ontario. Supplementary Fig. 4, Additional File 1 shows that the median LOS differs by province and triage level. The median LOS’s in Alberta generally increased over time.

### ED flow for 2015/2016

Our further examination of the effect of province and group on transitions among key stages of the ED presentation involved 549,266 and 655,199 presentations in the control and HSU Alberta groups, respectively, and 2,101,389 and 2,196,008 presentations in the control and HSU Ontario groups, respectively. For the presentations that flowed through the stages and completed care, patients in Alberta EDs took longer than those in Ontario when adjusted by group and triage level (Table 3). Patients in the HSU group had shorter times between start and PIA (HR = 1.03, 95% CI 1.02, 1.03) but had longer times to disposition decision and to end of the ED presentation than the control group (Decision: HR = 0.84, 95% CI 0.84, 0.84; End: HR = 0.91; 95% CI 0.91, 0.91). For patients who did not complete care, Albertans had shorter times to LWBS and LAMA than Ontarians. Notably, the HSU group also had shorter times in these stages than the control group (LWBS: HR = 1.41, 95% CI 1.40, 1.43; LAMA: 1.63, 95% CI 1.59, 1.67).

**Table 3:** Hazard ratios (HRs) and associated 95% confidence intervals (CIs) for flow between stages for presentations that ended with care completed and those that ended before care completed (LWBS = left without being seen, LAMA = left against medical advice) for fiscal year 2015/2016

**Presentations where ED Care was Completed**			
	**Start-Physician HR (95% CI) (*n*** **= 4,783,267)**	**Physician-Decision HR (95% CI) (*n*** **= 4,712,493)**	**Decision-End HR (95% CI) (*n*** **= 4,712,452)**
Province (reference = Ontario)	0.96 (0.96, 0.97)*	0.81 (0.81, 0.81)*	0.98 (0.98, 0.99)*
Group (reference = Control)	1.03 (1.02, 1.03)*	0.84 (0.84, 0.84)*	0.91 (0.91, 0.91)*
Triage level (reference = 5-Non-urgent)			
1 - Resuscitation	4.49 (4.45, 4.54)*	0.19 (0.19, 0.20)*	0.31 (0.30, 0.31)*
2 - Emergent	0.92 (0.92, 0.93)*	0.20 (0.20, 0.20)*	0.50 (0.50, 0.50)*
3 - Urgent	0.74 (0.73, 0.74)*	0.29 (0.29, 0.30)*	0.71 (0.71, 0.71)*
4 - Less-urgent (Semi-urgent)	0.88 (0.88, 0.88)*	0.70 (0.70, 0.71)*	0.96 (0.95, 0.96)*
**Presentations where Patient Left before Completion of Care**			
	**Start-LWBS HR (95% CI) (*n*** **= 146,461)**	**Physician-LAMA HR (95% CI) (*n*** **= 30,740)**	
Province (reference = Ontario)	1.06 (1.04, 1.07)*	1.06 (1.03, 1.09)*	
Group (reference = Control)	1.41 (1.40, 1.43)*	1.63 (1.59, 1.67)*	
Triage level (reference = 5-Non-urgent)			
1 - Resuscitation	0.11 (0.09, 0.15)*	0.41 (0.36, 0.47)*	
2 - Emergent	0.21 (0.20, 0.21)*	0.54 (0.50, 0.58)*	
3 - Urgent	0.36 (0.35, 0.37)*	0.65 (0.60, 0.70)*	
4 - Less-urgent (Semi-urgent)	0.59 (0.57, 0.60)*	0.84 (0.77, 0.91)*	

* denotes *p* < 0.05; *n* number of emergency department presentations

## Discussion

We examined over 38 million ED presentations in Alberta and Ontario made by the top 10% of the most frequent users and a sample of controls for each year (2011/2012 to 2015/2016) from a population-based database. The study described characteristics of the ED presentations and focused on key patient flow measures of time to PIA and LOS in the ED. To our knowledge, we are the first to compare HSUs and provinces on patient flow measures in the ED. EDs in Ontario and British Columbia were compared on flow measures for adults and children but did not consider frequent users of EDs [25]. Other studies have examined a single ED or jurisdiction to describe [26, 27] or compare [28–30] frequent users to other groups.

In our study, the Ontario groups had higher acuity than the Alberta groups. The median time to PIA remained relatively stable across all years and all acuity groups (e.g., 60 min to 67 min across provinces and groups in 2015/2016). When examined by acuity group, CTAS levels 2 to 5 had more similar median times in Ontario than compared to Alberta. Our modeling of patient flow data for 2015/2016, showed that HSUs had shorter times to PIA than controls (HR = 1.02) and Ontarians had longer times to PIA than Albertans (HR = 0.96), when adjusted by CTAS. These differences are statistically significant, although for individual patients such differences likely will not affect ED presentation outcomes.

Across all years and all acuity groups, our study showed that LOS remained relatively stable with Ontario groups having longer median LOS than Alberta groups, and the Alberta HSUs having the shortest LOS (3h6min Ontario HSUs, 2h48min for Ontario controls; 2h18min Alberta HSUs, 2h42min Alberta controls). In one suburban ED in Alberta in 2010/2011, 22,333 ED presentations were compared for non-frequent users (1–4 presentations), frequent users (≥5 presentations), and extreme frequent users (≥8 presentations) and the frequent users groups had longer mean LOS than the non-frequent user group [28]. The mean LOS values were 5h30min, 8 h, and 7h54min for the non-frequent, frequent, and extreme frequent user groups, respectively. These mean LOS values were longer than the control and HSU groups in our study. A study of 75, 141 patients with 98, 908 presentations to one ED and one minor injury unit in a city in the United Kingdom in 2003 showed that discharged frequent users had a mean LOS that was 40 min longer than discharged non-frequent users [30]. In our study, discharged HSUs in Alberta had about the same mean LOS as discharged controls whereas discharged HSUs in Ontario had a mean LOS about 12 min longer than discharged controls. A study in an urban ED in Ottawa, Ontario showed that 261 highly frequent ED users (patients in the 99th percentile during 2014) with 3164 presentations had a median LOS of 5.2 h (Q1, Q3 3.1, 8.7) [26] and a study in a Singapore ED showed that 243 frequent users (≥4 presentations in 2015) with 1705 presentations had a median LOS of 2h54min (Q1, Q3 1h42min, 5 h) [27] but neither study compare this measure to non-frequent users. These highly frequent ED users had longer median LOS than either of the HSU groups in our study and Ontario HSUs had about the same median as the Singapore frequent users. When examined by acuity group, our HSUs in the highest acuity groups had longer LOSs than controls. In 2015/2016, our modeling showed HSUs had longer times from assessment to decision and from decision to end when adjusted by CTAS (HR = 0.84 and HR = 0.91, respectively). In addition, Albertans had longer times from assessment to decision and from decision to end, when adjusted by CTAS.

HSU groups also had more ED presentations that were LWBS or LAMA compared to control groups. Other studies have shown that frequent ED users are more likely to have presentations that end in LWBS or LAMA [29, 31]. In 2015/2016, the model showed that HSUs had shorter times to LWBS and LAMA when adjusted by CTAS. Albertans had shorter times to LWBS and LAMA than Ontarians.

Taken together, HSUs longer stays in EDs following PIA, more LWBS and LAMA, and higher rates of admission suggest that HSUs are complex patients with health needs that EDs are not optimally equipped to manage. This finding supports interventions to link frequent ED users to alternate care settings as described by several previous studies [32–34].

Our study has several limitations. The data were obtained from paper-based sources and there may be some errors in the coding and the documented times that are provided to the population-based databases. In particular, we observed differential proportions of missing PIA times between provinces that may suggest bias in the assessment of time to PIA. Missing data on PIA times may occur when ED presentations end in LWBS, when time to PIA occurs after disposition date/time or patient left ED date/time, and for scheduled visits to the ED. Additionally, not all hospitals in Alberta report PIA to NACRS and rural EDs did not report PIA as regularly as less rural EDs. Calculations of the estimated median times incorporating censored times provided nearly identical values as the sample medians for the complete data. Second, our results may not be generalizable to other areas of Canada or other jurisdictions with different health care systems. Third, the cohorts defined by CIHI were based on unscheduled ED presentations. There are a few control patients in our study who may have had more presentations than the patients in the HSU group. Fourth, CIHI’s definition of HSU as the top 10% of ED presenters is relative to fiscal year and province, meaning individual patients may not meet the threshold in different years even if the same number of ED presentations are made. Fifth, other health services use data such as physician claims was unavailable so we cannot determine if HSUs of ED services are also HSUs of other health services. Also, other health services data could not be used to determine comorbidities. Sixth, important subgroups among HSUs do not appear through our analysis. Other studies have suggested bi-modal age distribution among HSUs [1], and differences between frequent users and extremely frequent users [1, 4, 28], Vaillancout [35] and colleagues have alternately argued for four distinct types or profiles of frequent users related to medical complexity/frailty, diagnostic uncertainty, converging medical and social issues, and those with serious recurring health conditions. Finally, we have assumed independence of ED presentations for the ED flow analysis.

## Conclusion

Ontario HSUs had higher acuity and longer lengths of stay than the other groups. In both provinces, HSUs had shorter times to PIA and longer times after that assessment. Further study is needed to identify subgroups of HSUs who might receive more appropriate care in alternate settings and interventions, in EDs and elsewhere, to link these HSUs to these settings. Reducing the number of ED presentations and the duration of these presentations by HSUs may also positively impact ED flow.

## Supplementary information

**Additional file 1.** Contains supplementary tables and figures as a Microsoft Word file

## Data Availability

Data is the property of the Canadian Institute for Health Information and the authors are not allowed to provide the data. Requests can be made for the same data from the Canadian Institute for Health Information for researchers who meet the criteria for access to confidential data. Researchers are welcome to inquire for further information at https://www.cihi.ca/en/make-a-data-request.

## Abbreviations

CI: Confidence interval
CIHI: Canadian Institute for Health Information
CTAS: Canadian Triage and Acuity Score
ED: Emergency department
h: Hour
HR: Hazard ratio
HSU: High system user
ICD-10-CA: International Classification of Diseases and Related Health Problems, Tenth Revision, The Canadian Enhancement of ICD-10
IQR: Interquartile range
km: Kilometre
LAMA: Left against medical advice
LOS: Emergency department length of stay
LWBS: Left without being seen
min: Minute
n: Count
N.A.: Not applicable
NACRS: National Ambulatory Care Reporting System
PIA: Physician initial assessment
Q1: First quartile (25th percentile)
Q3: Third quartile (75th percentile)
SD: Standard deviation
US: United States
