# Supplementary material for: Frequent users of emergency departments and patient flow in Alberta and Ontario, Canada: an administrative data study
Source: BMC Health Serv Res. 2020 Oct 12;20:938. doi: 10.1186/s12913-020-05774-6 (PMC7552544; doi:10.1186/s12913-020-05774-6)
Supplement: Supplementary file 1 — Additional file 1. Contains supplementary tables and figures as a Microsoft Word file [file 12913_2020_5774_MOESM1_ESM.docx]

**Additional File 1 for “Frequent users of emergency departments and patient flow in Alberta and Ontario, Canada: an administrative data study”**

Supplementary Table 1. Main diagnosis by province and group for 2015/2016 (n, %).

| Diagnosis Category | Alberta | | Ontario | |
| --- | --- | --- | --- | --- |
|  | Control  (n= 560,755) | HSU  (n= 686,817) | Control  (n= 2,113,961) | HSU  (n= 2,222,173) |
| ***Quan et al Categories^1^*** |  | | | |
| Myocardial infarction | 977 (0.2) | 583 (0.1) | 3,487 (0.2) | 2,344 (0.1) |
| Congestive heart failure | 1,958 (0.3) | 4,352 (0.6) | 8,005 (0.4) | 20,930 (0.9) |
| Peripheral vascular disease | 331 (0.1) | 420 (0.1) | 1,576 (0.1) | 2,148 (0.1) |
| Stroke | 3,947 (0.7) | 2,825 (0.4) | 20,682 (1.0) | 16,584 (0.7) |
| Dementia | 581 (0.1) | 519 (0.1) | 1,892 (0.1) | 3,030 (0.1) |
| Chronic pulmonary disease | 10,542 (1.9) | 16,118 (2.3) | 36,371 (1.7) | 60,279 (2.7) |
| Rheumatic disease | 240 (0.0) | 533 (0.1) | 853 (0.0) | 1,346 (0.1) |
| Peptic ulcer disease | 437 (0.1) | 471 (0.1) | 1,240 (0.1) | 1,408 (0.1) |
| Mild liver disease | 241 (0.0) | 741 (0.1) | 758 (0.0) | 2,247 (0.1) |
| Moderate or Severe liver disease | 112 (0.0) | 458 (0.1) | 295 (0.0) | 1,664 (0.1) |
| Diabetes (with and without chronic complication) | 2,415 (0.4) | 5,213 (0.8) | 8,649 (0.4) | 19,468 (0.9) |
| Hemiplegia or paraplegia | 80 (0.0) | 119 (0.0) | 208 (0.0) | 370 (0.0) |
| Renal disease | 196 (0.0) | 569 (0.1) | 642 (0.0) | 1,661 (0.1) |
| Any malignancy, including lymphoma and leukemia, except malignant neoplasm of skin (Cancer) | 1,190 (0.2) | 2,280 (0.3) | 4,090 (0.2) | 9,437 (0.4) |
| Metastatic solid tumor | 316 (0.1) | 444 (0.1) | 1,274 (0.1) | 2,855 (0.1) |
| Acquired immunodeficiency syndrome/  human immunodeficiency virus (AIDS/HIV) | 11 (0.0) | 99 (0.0) | 57 (0.0) | 133 (0.0) |
| ***Guttmann et al. Adapted Categories^2^*** |  | | | |
| Injury or trauma, excluding rheumatic disease above | 192,261 (34.3) | 130,798 (19.0) | 750,984 (35.5) | 487,382 (21.9) |
| Mental health, excluding dementia above | 17,171 (3.1) | 40,983 (6.0) | 60,848 (2.9) | 147,739 (6.6) |
| Headache and other neurological conditions, excluding stroke, dementia, and hemiplegia/paraplegia above | 28,039 (5.0) | 30,766 (4.5) | 116,588 (5.5) | 112,548 (5.1) |
| Gastrointestinal | 29,614 (5.3) | 36,963 (5.4) | 103,734 (4.9) | 137,569 (6.2) |
| Chest pain | 23,569 (4.2) | 16,342 (2.4) | 105,274 (5.0) | 75,852 (3.4) |
| Shortness of breath | 2,871 (0.5) | 3,690 (0.5) | 12,939 (0.6) | 19,965 (0.9) |
| Upper respiratory infections/ Otitis media | 23,857 (4.3) | 18,869 (2.7) | 87,893 (4.2) | 69,283 (3.1) |
| Fever | 1,375 (0.2) | 1,316 (0.2) | 7,564 (0.4) | 9,311 (0.4) |
| Skin problems | 22,974 (4.1) | 31,562 (4.6) | 86,170 (4.1) | 112,545 (5.1) |
| Genitourinary/obstetric, excluding renal disease above | 46,162 (8.2) | 55,810 (8.1) | 159,960 (7.6) | 199,104 (9.0) |
| General signs and symptoms (K08, R53, R00, K62, R68, K59, K92, I10, or R60) | 18,452 (3.3) | 20,888 (3.0) | 77,039 (3.6) | 90,132 (4.1) |
| ***Not Defined in Either Coding Scheme*** |  | | | |
| Other | 130,836 (23.3) | 263,086 (38.3) | 454,889 (21.5) | 614,839 (27.7) |

HSU = high system users; n = number of emergency department presentations

1. Quan H, Sundararajan V, Halfon P, et al. Coding algorithms for defining comorbidities in ICD-9-CM and ICD-10 administrative data. Medical Care. 2005; 43:1130-9.
2. Guttmann A, Schull MJ, Vermeulen MJ, Stukel TA. Association between waiting times and short term mortality and hospital admission after departure from emergency department: Population based cohort study from Ontario, Canada. BMJ. 2011;342d2983.

Supplementary Table 2. Length of stay (LOS) in the ED for patient subgroups by province and group for 2015/2016.

|  |  | Alberta | | Ontario | |
| --- | --- | --- | --- | --- | --- |
|  |  | Control | HSU | Control | HSU |
| All Patients | | (n=560,755) | (n=686,817) | (n=2,113,961) | (n=2,222,173) |
|  |  |  |  |  |  |
|  | Median (Q1, Q3)* | 2.6 (1.4, 4.7) | 2.2 (1.1, 4.5) | 2.8 (1.6, 4.7) | 3.0 (1.6, 5.4) |
|  | Missing (%) | 13,395 (2.4) | 34,784 (5.1) | 6,922 (0.3) | 17,195 (0.8) |
|  | Estimated median time (95% confidence interval) † | 2.7 (2.7, 2.7) | 2.3 (2.3, 2.3) | 2.8 (2.8, 2.8) | 3.0 (3.0, 3.0) |
| Discharged | | (n=478,332) | (n=575,360) | (n=1,826,261) | (n=1,789,388) |
|  |  |  |  |  |  |
|  | Median (Q1, Q3)* | 2.4 (1.3, 4.0) | 1.9 (1.0, 3.8) | 2.5 (1.5, 4.1) | 2.6 (1.4, 4.4) |
|  | Missing (%) | 12,187 (2.5) | 31,652 (5.5) | 5,134 (0.3) | 13,631 (0.8) |
|  | Estimated median time (95% confidence interval) † | 2.4 (2.4, 2.5) | 2.0 (2.0, 2.0) | 2.6 (2.6, 2.6) | 2.6 (2.6, 2.6) |
| Admitted/Transferred | | (n=61,581) | (n=81,525) | (n=221,390) | (n=336,113) |
|  |  |  |  |  |  |
|  | Median (Q1, Q3)* | 8.2 (4.5, 15.3) | 7.3 (3.7, 14.4) | 9.3 (5.4, 17.8) | 9.3 (5.2, 18.4) |
|  | Missing (%) | 182 (0.3) | 485 (0.6) | 128 (0.1) | 467 (0.1) |
|  | Estimated median time (95% confidence interval) † | 8.3 (8.3, 8.4) | 7.5 (7.4, 7.5) | 9.4 (9.3, 9.4) | 9.4 (9.3, 9.4) |

HSU = high system users; SD = standard deviation; Q1 = 25^th^ percentile; Q3 = 75^th^ percentile; n = number of emergency department presentations;

* = missing times removed from calculation; † = all data used and missing times interval censored

Supplementary Figure 1. Flow through the ED (LWBS=left without being seen, LAMA=left against medical advice).


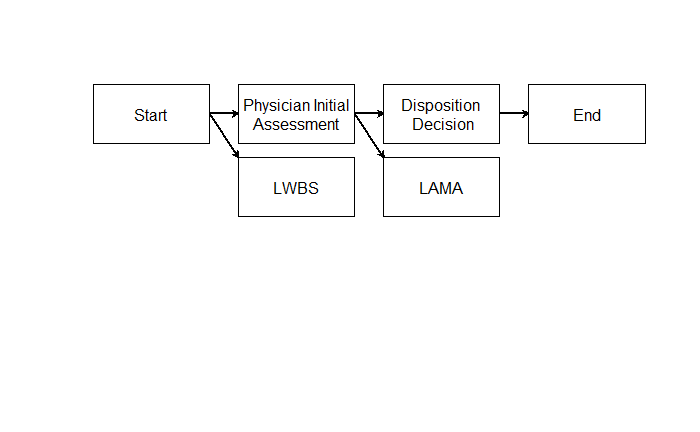


Supplementary Figure 2. Triage levels by province, group, and fiscal year.

| (a) Alberta, control group | (b) Alberta, HSU group |
| --- | --- |
| 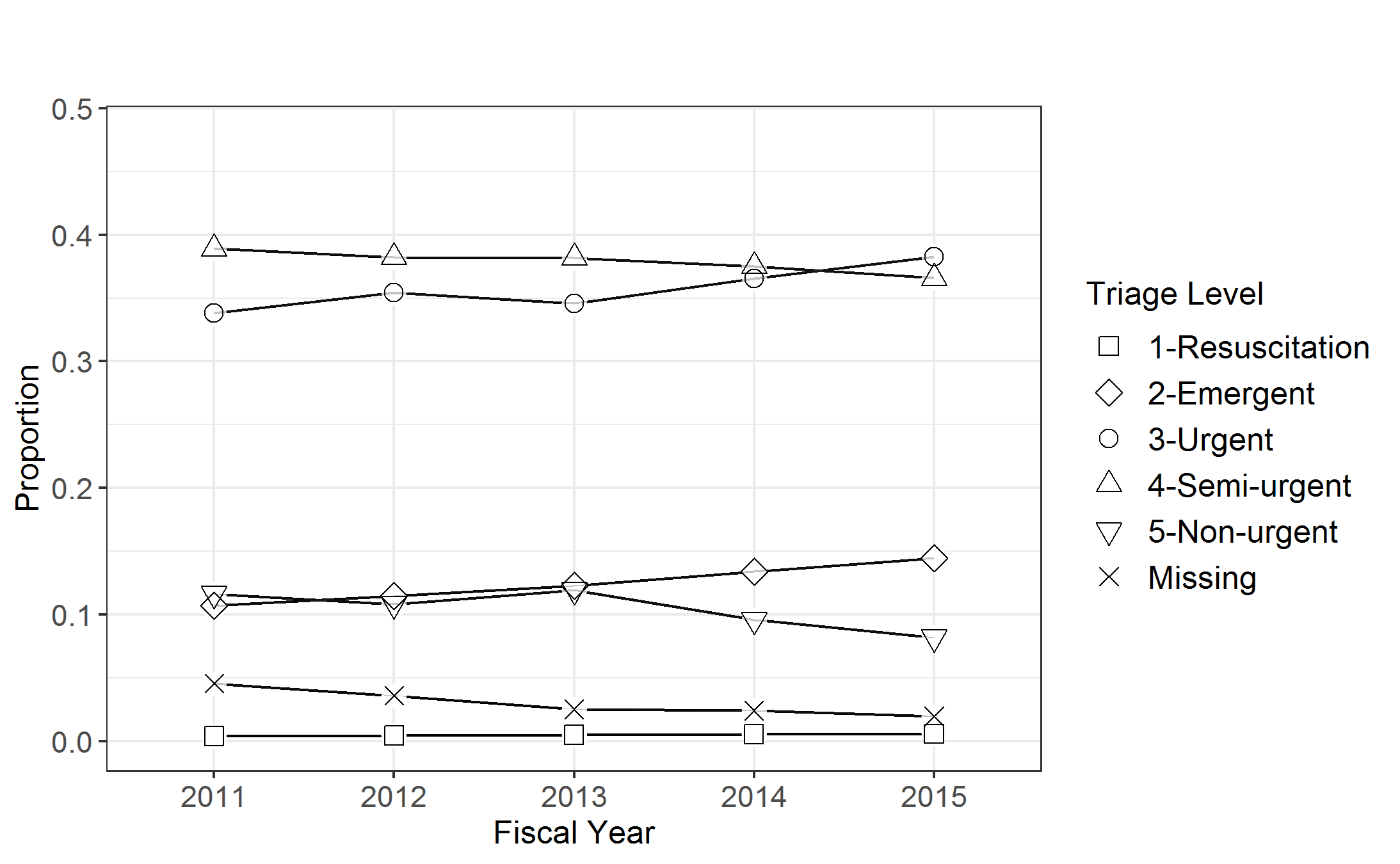 | 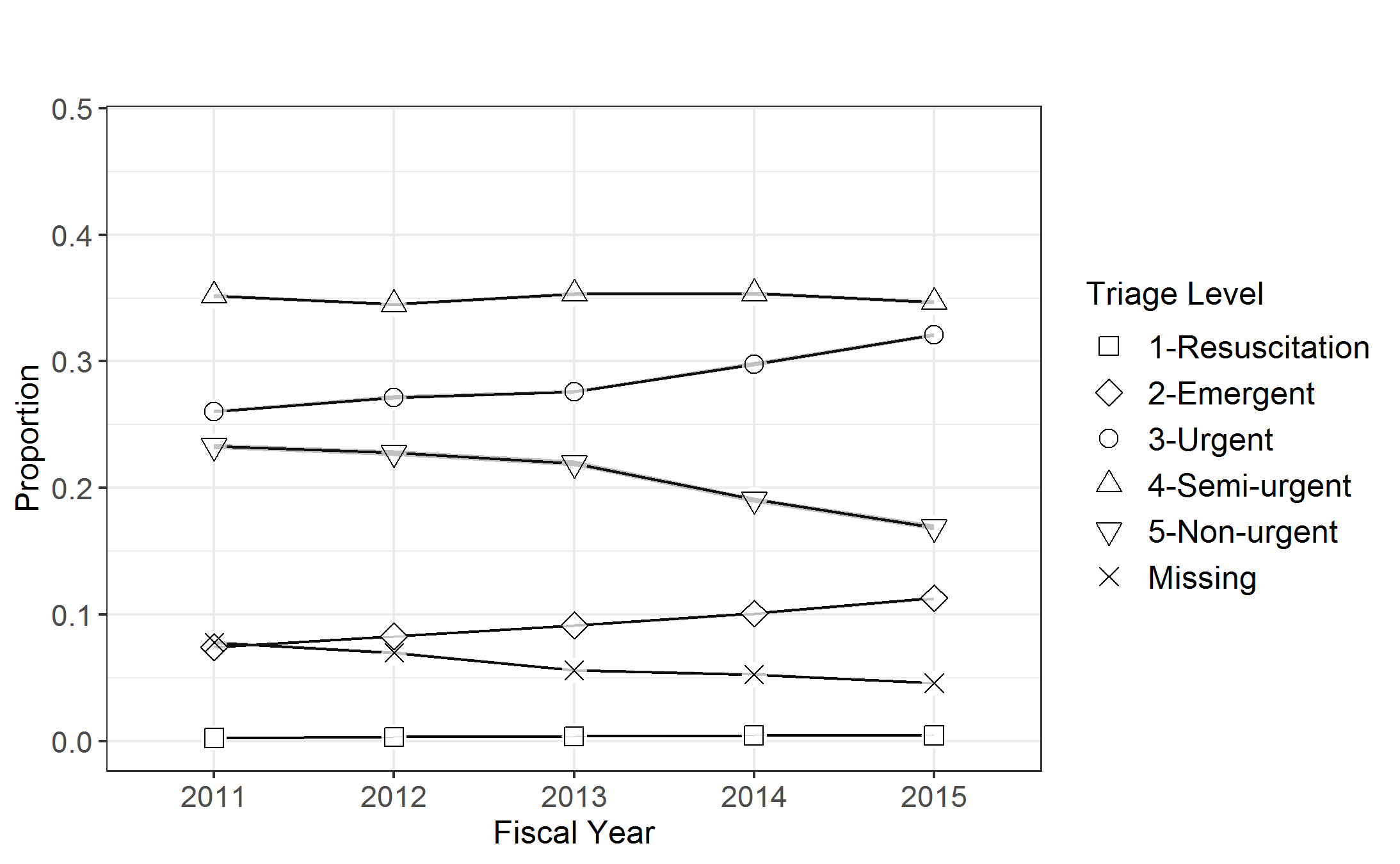 |
|  |  |
| (c) Ontario, control group | (d) Ontario, HSU group |
| 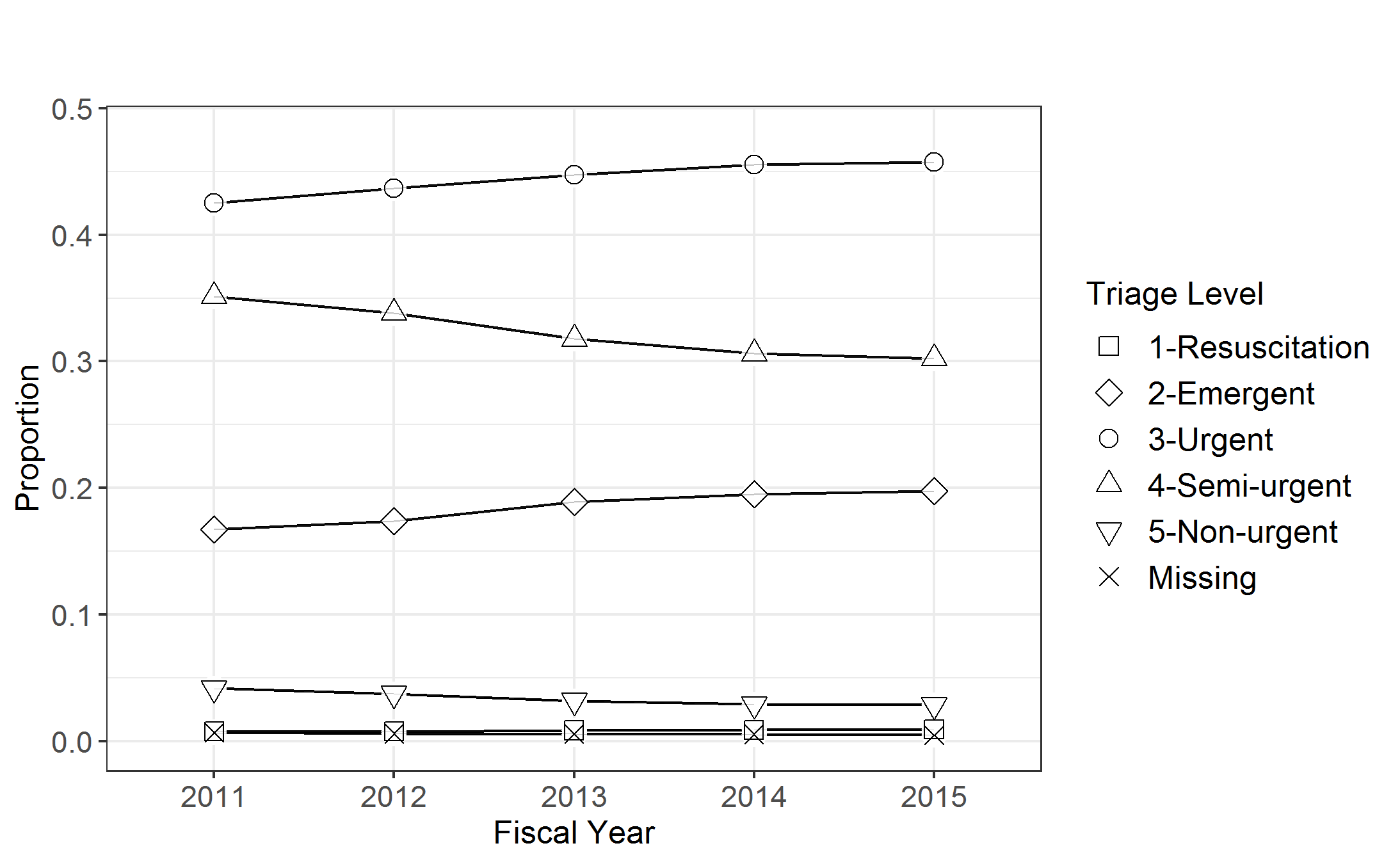 | 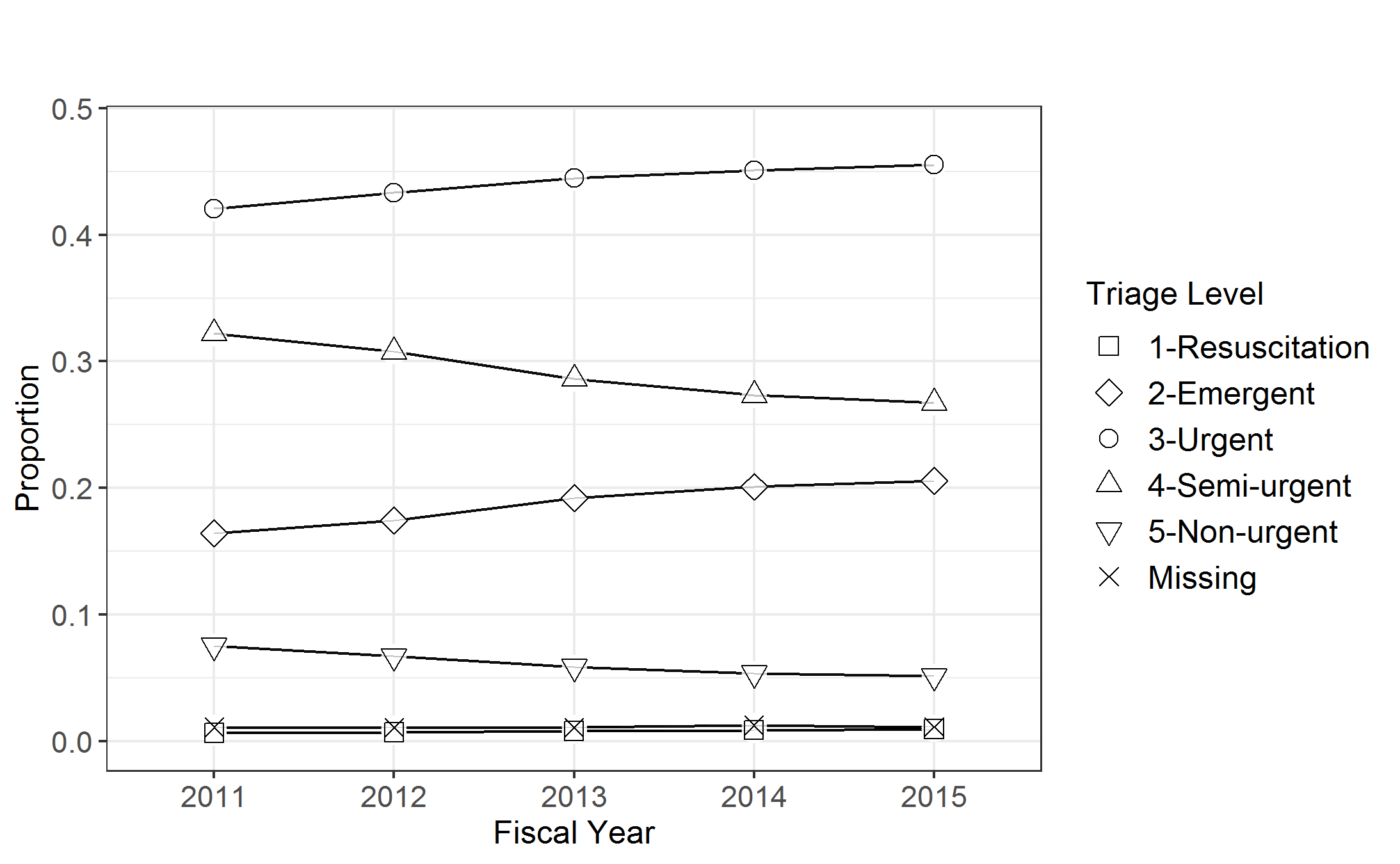 |

Supplementary Figure 3. Median ED length of stay for visits ending in discharge by triage level, province, group, and fiscal year.

| (a) Alberta, control group | (b) Alberta, HSU group |
| --- | --- |
| 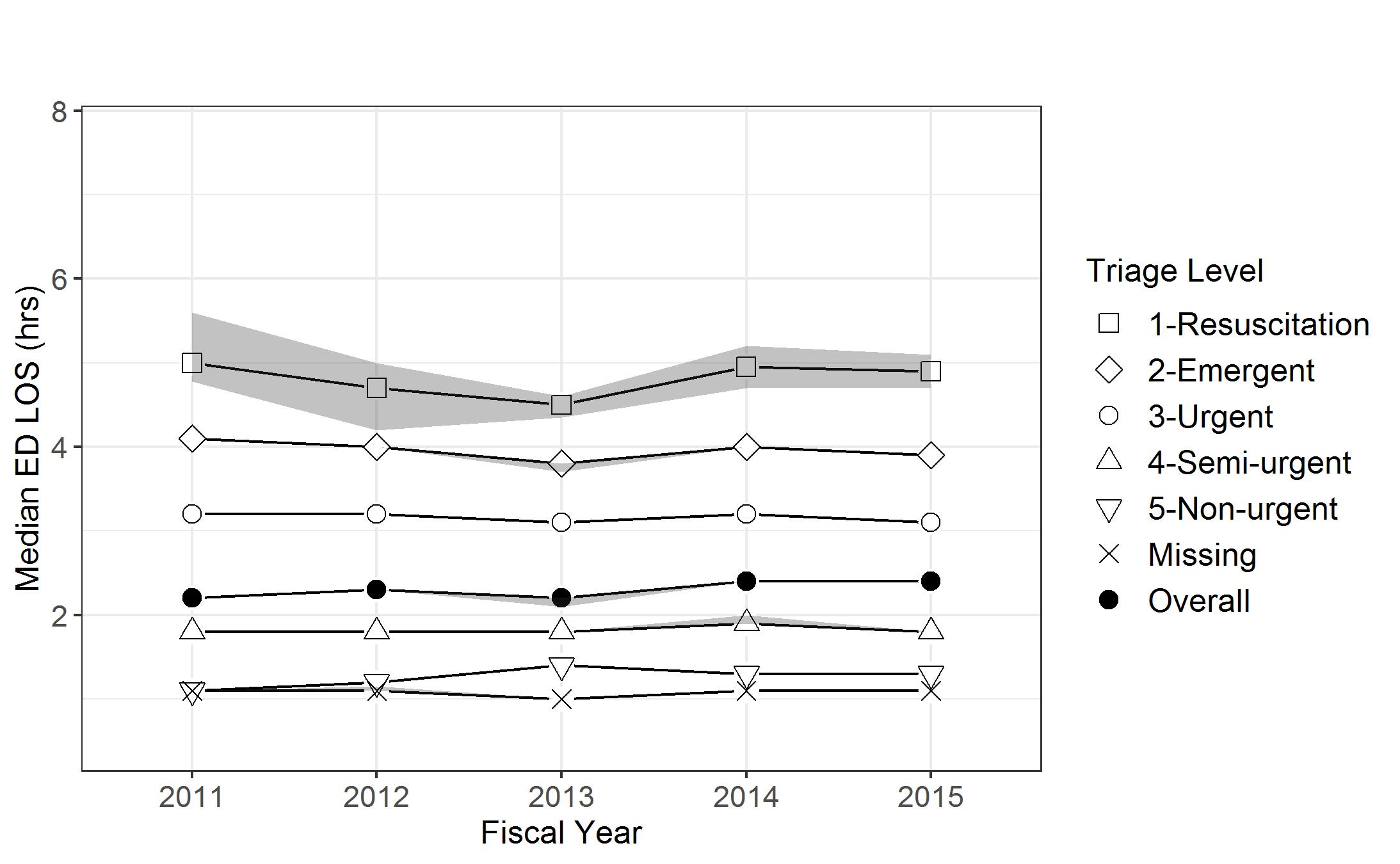 | 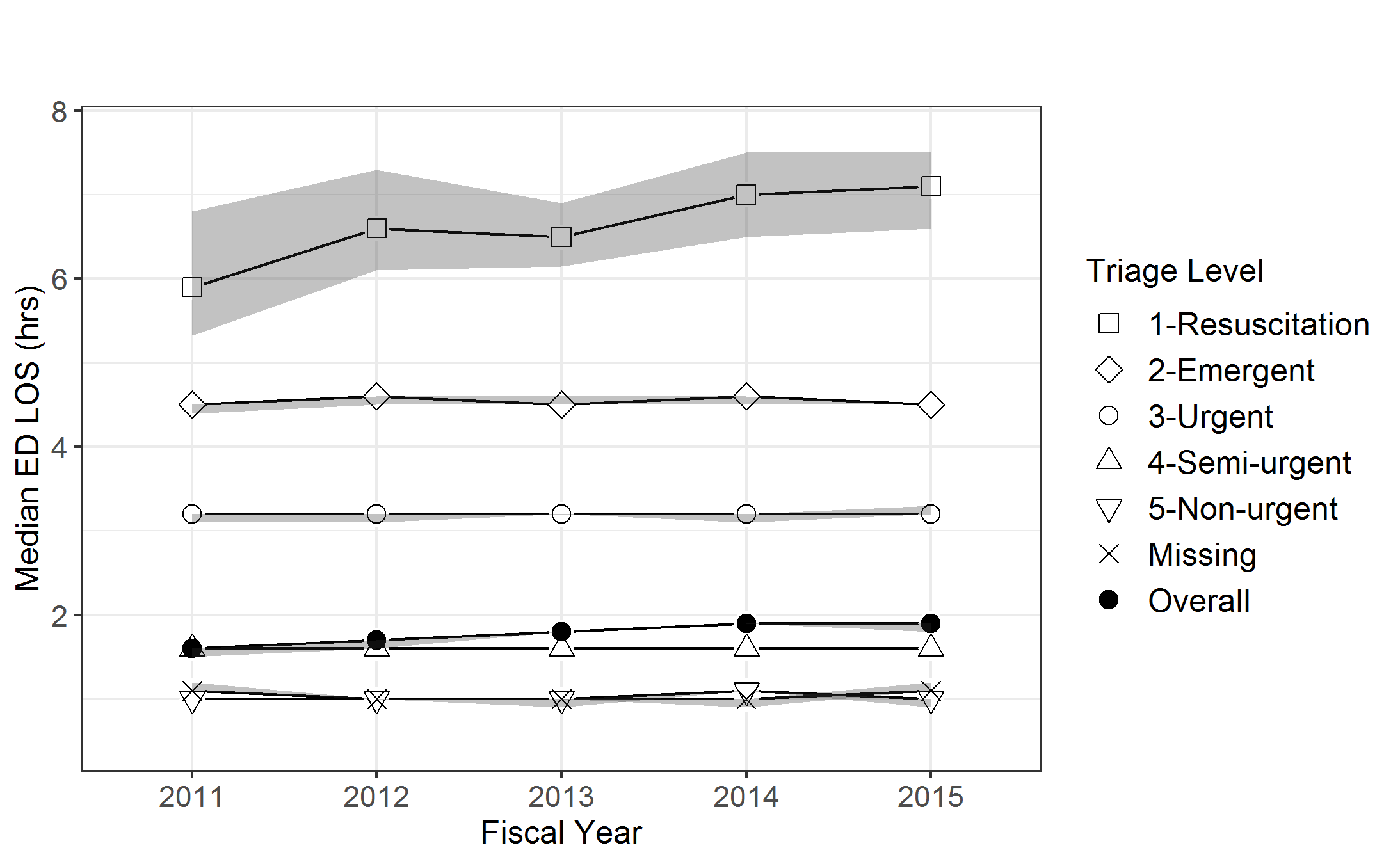 |
|  |  |
| (c) Ontario, control group | (d) Ontario, HSU group |
| 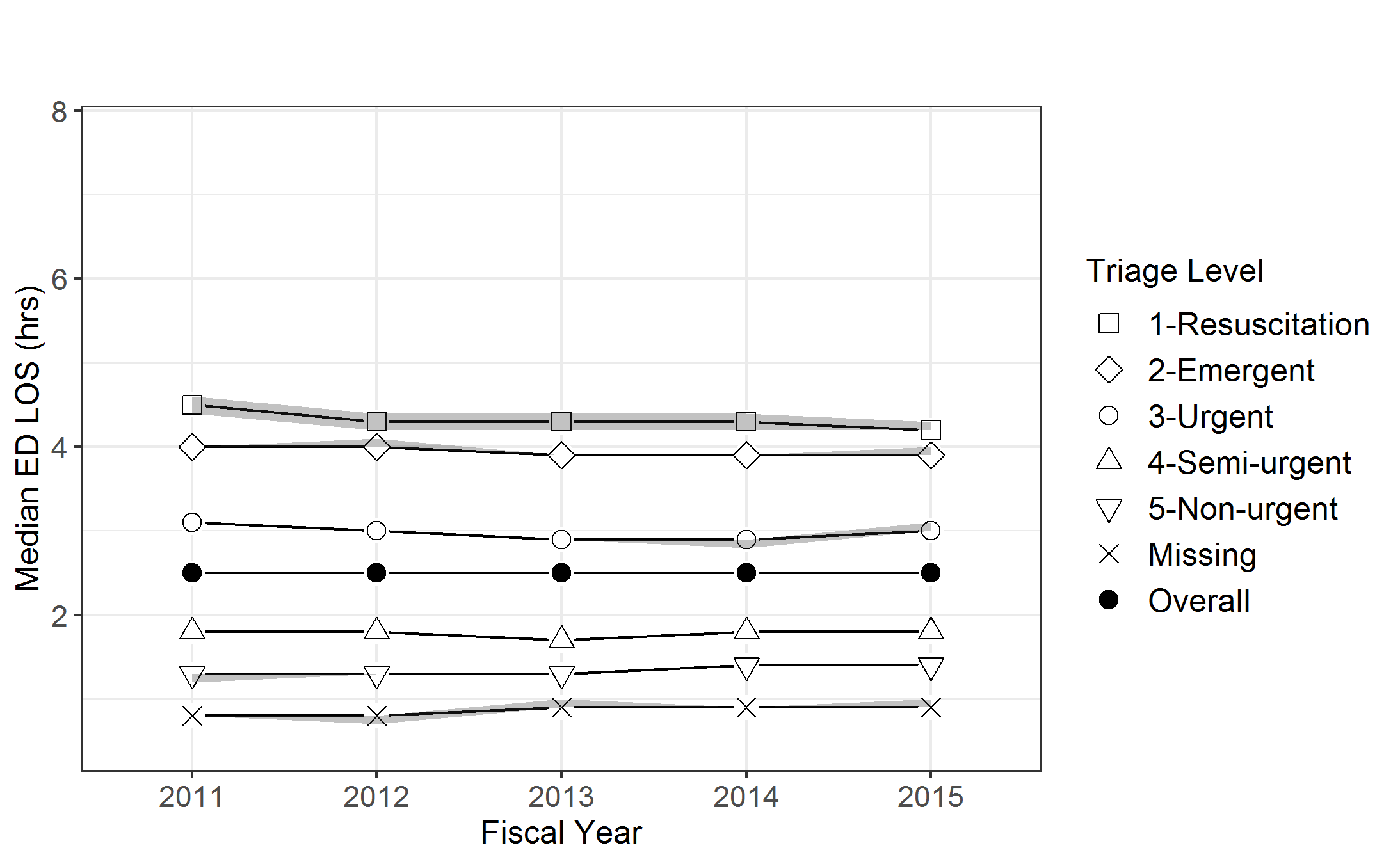 | 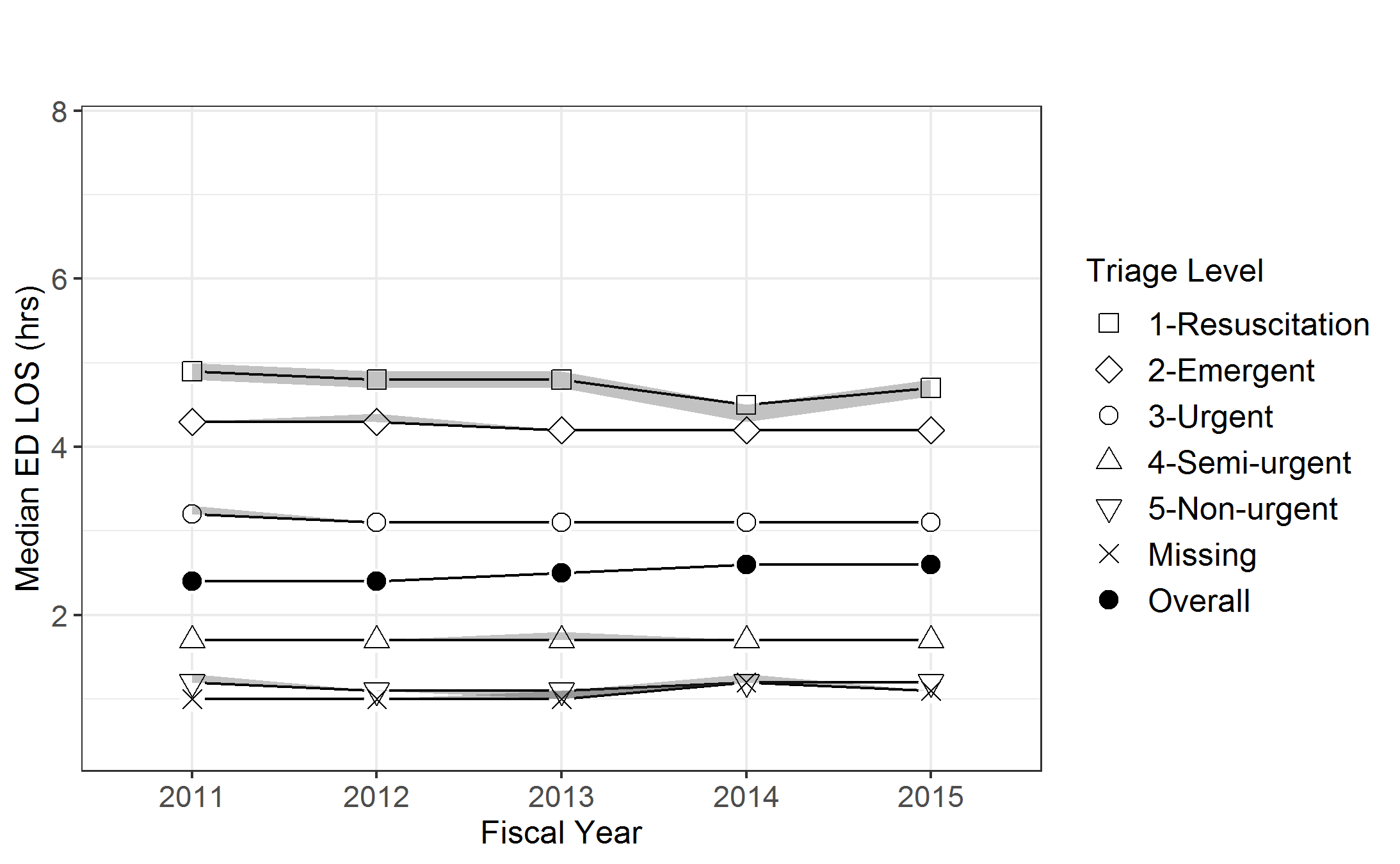 |

Supplementary Figure 4. Median ED length of stay for visits ending in admission or transfer by triage level, province, group, and fiscal year.

| (a) Alberta, control group | (b) Alberta, HSU group |
| --- | --- |
| 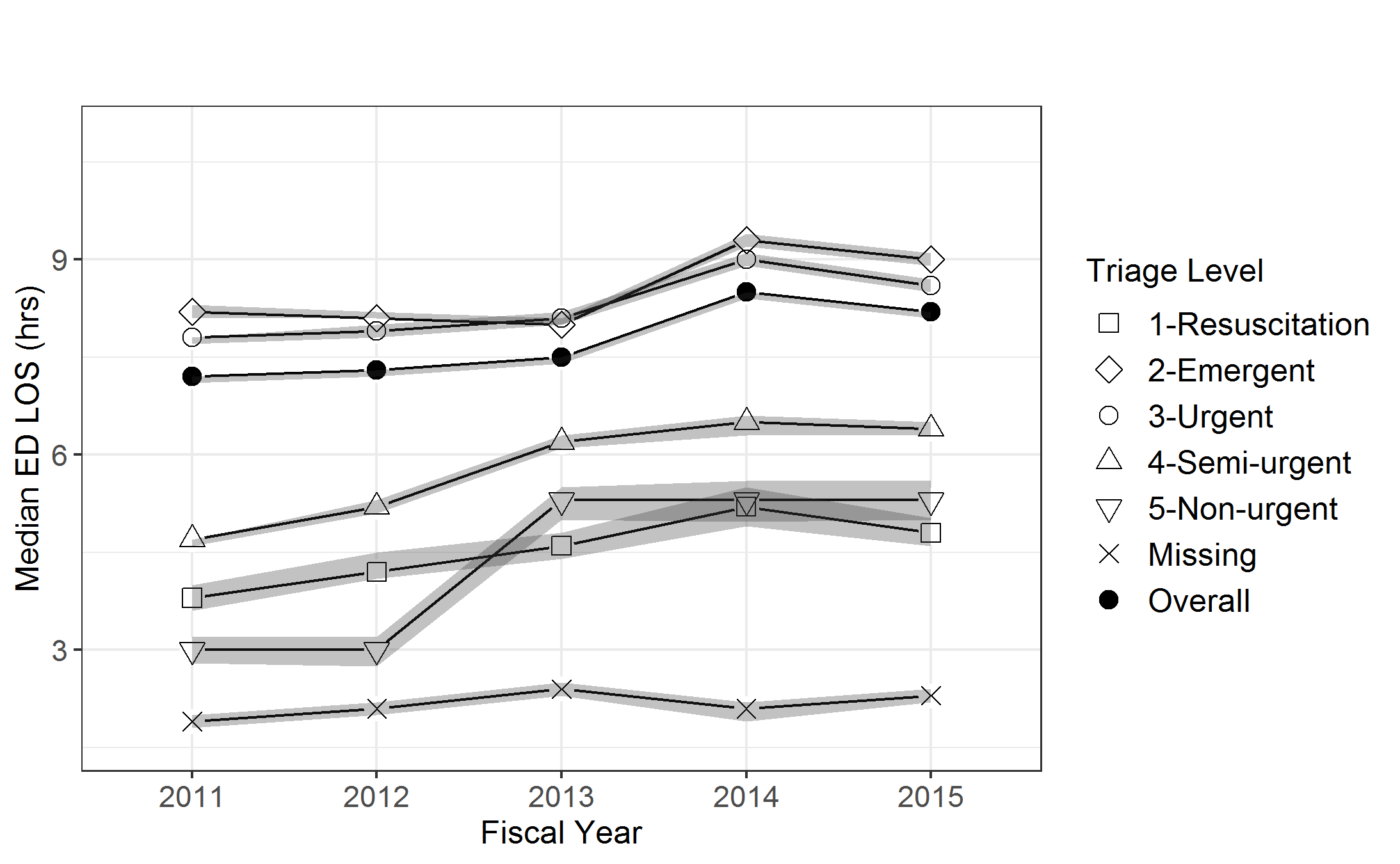 | 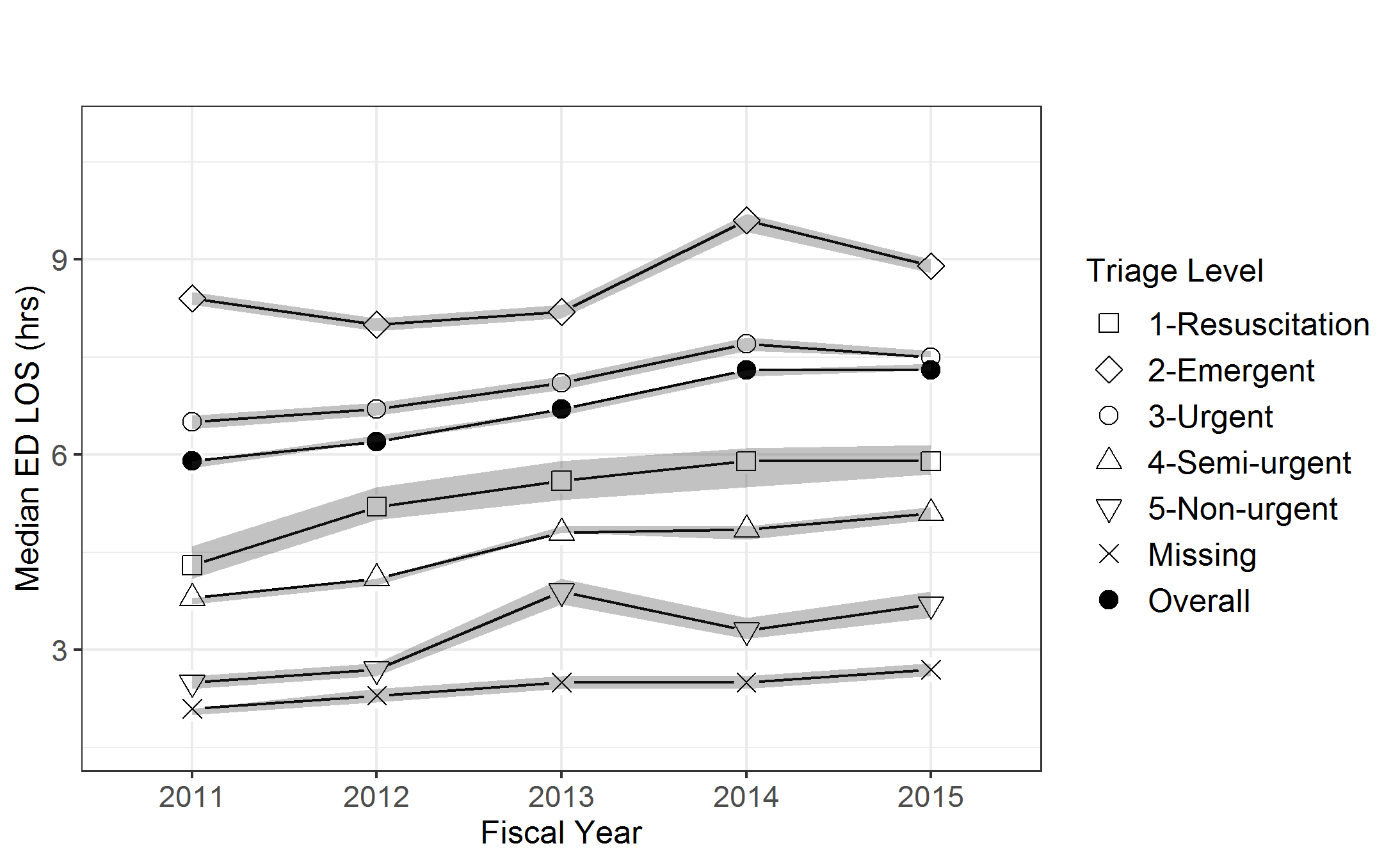 |
|  |  |
| (c) Ontario, control group | (d) Ontario, HSU group |
| 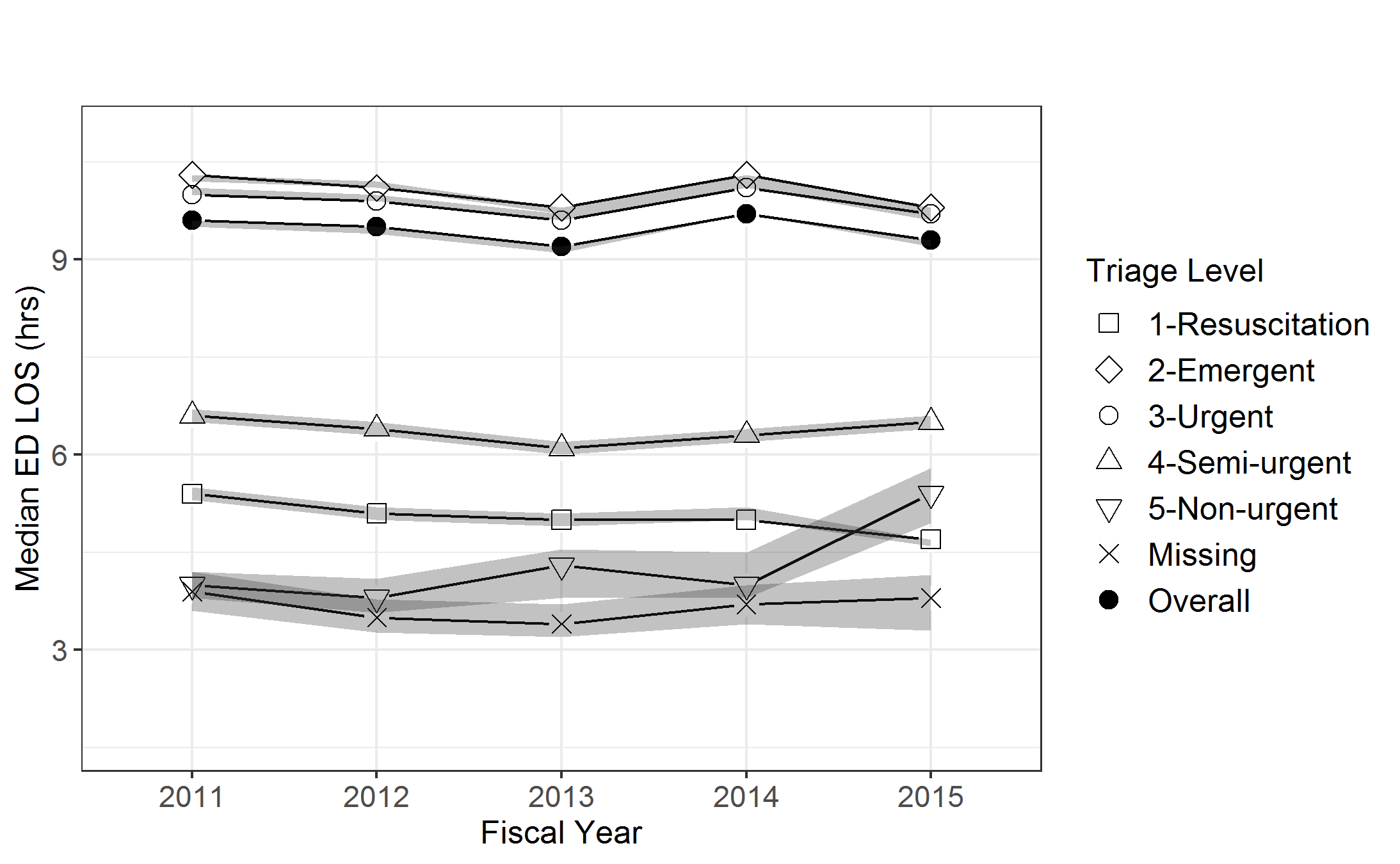 | 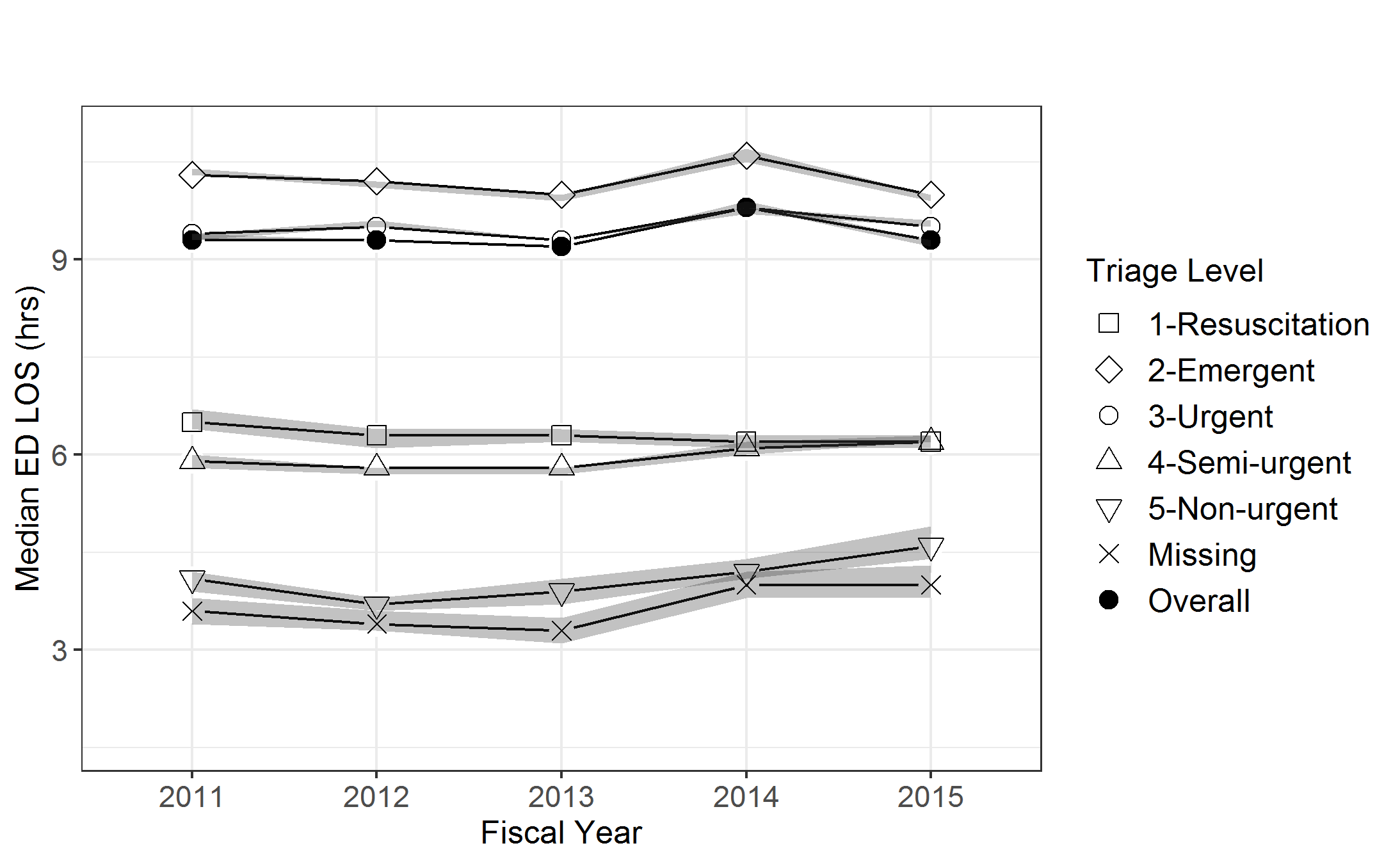 |
